# Characterisation of FUT4 and FUT6 α-(1→2)-Fucosyltransferases Reveals that Absence of Root Arabinogalactan Fucosylation Increases Arabidopsis Root Growth Salt Sensitivity

**DOI:** 10.1371/journal.pone.0093291

**Published:** 2014-03-25

**Authors:** Theodora Tryfona, Tina E. Theys, Tanya Wagner, Katherine Stott, Kenneth Keegstra, Paul Dupree

**Affiliations:** 1 School of Biological Sciences, Department of Biochemistry, University of Cambridge, Cambridge, United Kingdom; 2 DOE Plant Research Laboratory, Michigan State University, East Lansing, Michigan, United States of America; 3 School of Biological Sciences, Department of Biochemistry, University of Cambridge, Cambridge, United Kingdom; Wake Forest University, United States of America

## Abstract

Plant type II arabinogalactan (AG) polysaccharides are attached to arabinogalactan proteins (AGPs) at hydroxyproline residues, and they are very diverse and heterogeneous structures. The AG consists of a *β*-(1→3)-linked galactan backbone with *β*-(1→6)-galactan side chains that are modified mainly with arabinose, but they may also contain glucuronic acid, rhamnose or other sugars. Here, we studied the positions of fucose substitutions in AGPs, and we investigated the functions of this fucosylation. Monosaccharide analysis of Arabidopsis leaf AGP extracts revealed a significant reduction in L-Fucose content in the *fut4* mutant, but not in the *fut6* mutant. In addition, Fucose was reduced in the *fut4* mutant in root AGP extracts and was absent in the *fut4/fut6* mutant. Curiously, in all cases reduction of fucose was accompanied with a reduction in xylose levels. The fucosylated AGP structures in leaves and roots in wild type and *fut* mutant plants were characterised by sequential digestion with AG specific enzymes, analysis by Polysaccharide Analysis using Carbohydrate gel Electrophoresis, and Matrix Assisted Laser Desorption/Ionisation (MALDI)-Time of Flight Mass spectrometry (MS). We found that FUT4 is solely responsible for the fucosylation of AGPs in leaves. The *Arabidopsis thaliana FUT4* and *FUT6* genes have been previously proposed to be non-redundant AG-specific fucosyltransferases. Unexpectedly, FUT4 and FUT6 enzymes both fucosylate the same AGP structures in roots, suggesting partial redundancy to each other. Detailed structural characterisation of root AGPs with high energy MALDI-Collision Induced Dissociation MS and NMR revealed an abundant unique AG oligosaccharide structure consisting of terminal xylose attached to fucose. The loss of this structure in *fut4/fut6* mutants explains the reduction of both fucose and xylose in AGP extracts. Under salt-stress growth conditions the *fut4/fut6* mutant lacking AGP fucosylation exhibited a shorter root phenotype than wild type plants, implicating fucosylation of AGPs in maintaining proper cell expansion under these conditions.

## Introduction

Proteins glycosylated with arabinogalactan (AGPs) have been implicated in growth and a wide variety of developmental processes [1], [2], [3]. The arabinogalactan (AG) may influence cell surface protein trafficking and stability, perform functions such as chaperoning polysaccharides or Ca^+^ chelation, and it may generate signalling molecules [4], [5].

The AGs can be large branched polysaccharides and may consist of as many as 100 to 150 sugar residues [6], [7]. The AGs show vast heterogeneity not only in size but also in composition and structure. The AG consists of a *β*-(1→3)-galactan backbone with *β*-(1→6)-linked galactan side chains modified by *α*-(1→3)-L-arabinosyl (L-Ara) residues (known as type II AG; [8], [9]. Tan *et al.*, have reported an alternative modular structure for AGPs heterologously expressed in tobacco (*Nicotiana tabacum*; [10], consisting of repeating blocks of 15 sugar residues of decorated *β*-(1→3)-trigalactosyl subunits connected with *β*-(1→6)-linkages. In both models, the *β*-(1→6)-galactan side chains can be further modified with other less abundant sugars such as glucuronic acid (GlcA) and its 4-*O*-methylated counterpart (4-*O*-Me-GlcA), L-rhamnose (Rha) and L-fucose (Fuc) [7], [9], [11], [12], [13]. Recently GlcA or 4-*O*-Me-GlcA modifications of the *β*-(1→3)-galactan backbone were reported [14]. The AGP APAP-1 from Arabidopsis is reported to have short arabinoxylan and pectin side chains [15]. However, any function of the different sugar moieties on the AG are unclear.

Radish leaf AGPs are modified with Fuc residues [16] but this sugar is missing from radish roots [9]. Fuc is reported in AGPs of Arabidopsis leaves (*Arabidopsis thaliana*; [14], radish [16], [17], [18], thyme (*Thymus vulgaris*; [19] and celery (*Apium graveolens*; [20]. In Arabidopsis and radish leaf AGPs, Fuc has been reported in the structure α-L-Fuc-(1→2)-α-D-Ara-(1→) [14], [17]. The biological roles of fucosylation however, remain unclear although there are some lines of evidence suggesting that extracellular Fuc may be involved in plant cell signalling and development: Fuc has been detected in root exudates from maize (*Zea mays*; [21]) and wheat (*Triticum aestivum* L.; [22]). Experiments using Fuc specific lectins or fucosidases have suggested that this sugar may play a role in root-microbe interactions [23]. In addition, the Arabidopsis *mur1 (murus 1)* mutant which is affected in the biosynthesis of Fuc has been reported to lack Fuc from the cell wall polymers rhamnogalacturonan-II (RG-II) and xyloglucan (XG) [24], as well as from leaf AGPs [14]. *Mur1* plants were slightly dwarfed and the mechanical strength of the cell wall was reduced and they had shorter roots compared to wild-type [25]. Root AGP preparations were found to have 40% less Fuc than wild-type plants [25]. Addition of the Fuc-binding eel lectin or the AG *β*-(1→3)-galactan backbone reactive β-Glc-Yariv reagent [26] to the growth medium almost completely phenocopied *mur1*, suggesting that fucosylated root AGPs may play a role in root elongation. However, Fuc is present in several plant glycans, including xyloglucan, RG-I, RG-II and some N-glycans, complicating the interpretation of the *mur1* phenotype.

Two members of the plant GT37 family (FUT4 and FUT6) have been biochemically characterised to transfer Fuc to AG *in vitro* [27]. It was proposed that they have different fucosyl transfer activities, but it remains unclear what fucosylated structures were generated by these enzymes. The phenotype of the corresponding *fut* mutants has not been reported. One of the major bottlenecks in identifying the roles of AGP biosynthetic glycosyltransferases is the difficulty of characterising changes in AG structure in putative mutants. Recently, we reported a powerful approach for the structural characterisation of the carbohydrate components of AGPs which combines the use of AG-specific enzymes, high-energy matrix assisted laser desorption/ionisation (MALDI)-collision induced dissociation (CID) mass spectrometry (MS) and polysaccharide analysis using carbohydrate gel electrophoresis (PACE; [7]). This technique allowed the characterisation of the structure of Arabidopsis leaf AG polysaccharides and the Fuc modifications on those molecules [14].

In this work, we characterised the structure of AGPs to determine the function of AtFUT4 and AtFUT6 in the synthesis of Arabidopsis leaf and root fucosylated AGPs, and show that complete loss of Fuc in root AGPs leads to increased salt sensitivity in root growth.

## Results

### Identification of T-DNA Insertion Mutants

**Figure 1:**
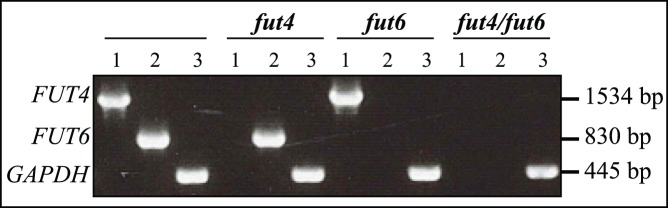
RNA transcript levels of *FUT4* and *FUT6* genes by RT-PCR. RNA was isolated from roots 14 days after germination from homozygous *fut4*, *fut6*, *fut4/fut6* and wild-type seedlings and RT-PCR was performed using the primers listed in Table S1. Glyceraldehyde 3-phosphate dehydrogenase (GAPDH) was used as a control. In lane 1 the *fut4* left (LP) and right (RP) primers were used. In lanes 2 and 3, the *fut6* and *gapdh* primers (LP and RP) respectively, were used.

To investigate the functional roles of AtFUT4 and AtFUT6, T-DNA insertional lines were identified (Figure S1 a and b). Arabidopsis plant lines *fut4* (SAIL_284_B05) and *fut6* (SALK_078357) homozygous for the insertion were identified by PCR analysis (Figure S1 a and b). The morphology of the *fut4* and *fut6* plants appeared similar to the wild-type plants (this is discussed later) and hence a *fut4/fut6* double mutant was generated (Figure 1). *FUT4* transcripts were not detected in the homozygous mutant by RT-PCR. Similarly, absence of *FUT6* transcript in the corresponding mutant was confirmed, and no *FUT4* and *FUT6* transcripts were detected in the double mutant (Figure 1).

### AGPs from Arabidopsis *fut4* Leaves Lack Fuc Modifications

**Figure 2:**
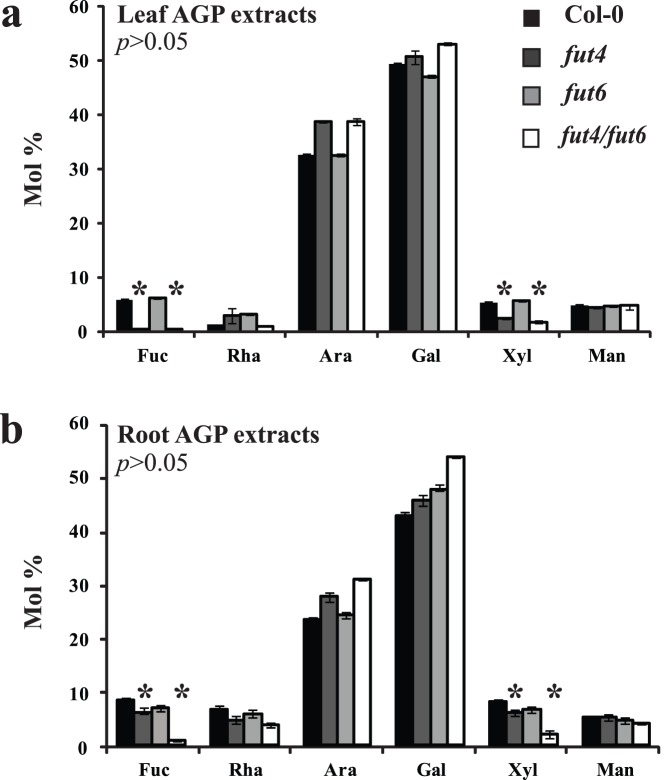
HPAEC-PAD monosaccharide analysis of neutral sugars of AGP extracts from Arabidopsis wild-type, *fut4*, *fut6* and *fut4/fut6* double mutant (a) leaves and (b) roots. Bars represent mean ± SD (n = 3). A significant difference was identified between wild type and *fut4* single- and *fut4/fut6* double-mutant plants for Fuc and Xyl sugars both in leaves and roots as indicated by *p*>0.05 in students *t-*test.

AtFUT4 is expressed in all tissues but has higher levels of transcript accumulation in leaves, although it is also expressed in roots [28]. AtFUT6 on the other hand, is expressed in roots but not leaves [28]. To determine any changes in AGP sugar composition in the *fut4, fut6* and *fut4/fut6* mutants, AGP-enriched extracts were prepared from both leaves and roots and their monosaccharide content was analysed by High pH Anion Exchange Chromatography (HPAEC) with Pulse Amperometric Detection (PAD; Figure 2). High Ara and Gal amounts were detected in both leaf and root samples, consistent with the high AGP content of the sample. Significant differences in neutral sugar composition were found between the mutants and the wild-type samples from both leaves (Figure 2a) and roots (Figure 2b). Fuc was absent in AGP extracts from *fut4* mutant leaves and was also reduced in *fut4* root AGP extracts. The double *fut4/fut6* mutant contained no detectable Fuc in leaf and very low levels of Fuc in root extracts. These results are consistent with a role for FUT4 in AGP fucosylation in leaves and a role for both FUT4 and FUT6 enzymes in roots. Interestingly, the amount of Xyl was reduced in AGP extracts from *fut4* and *fut4/fut6* leaves and from *fut4/fut6* roots. To confirm that the compositional changes in leaf AGP extracts is due to the *fut4* mutation, the *FUT4* gene was reintroduced into the corresponding *fut4* mutant. The decreases in Fuc and Xyl in AGP extracts were reversed by complementation of *fut4* with the wild type *FUT4* gene (Table S2).

**Figure 3:**
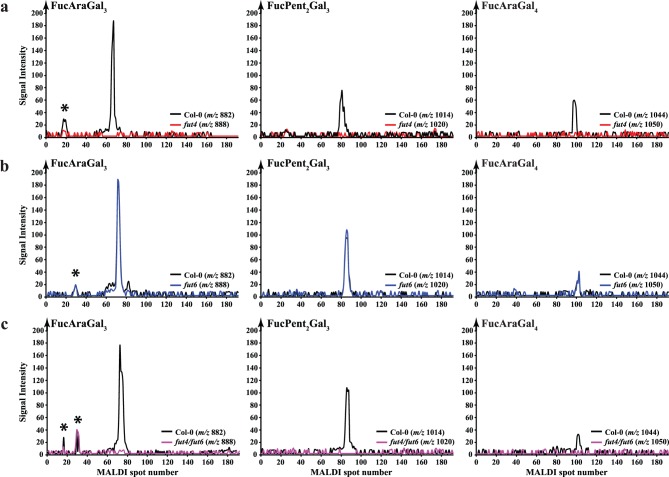
Capillary HILIC-MALDI-ToF-MS using stable isotope tagging of fucosylated oligosaccharides from Arabidopsis leaf AGP extracts of Columbia wild-type (Col-0; black line), *fut4* (red line), *fut6* (blue line) and *fut4/fut6* (magenta line) plants. Purified leaf AGP extracts were subjected to sequential digestion with α-arabinofuranosidase, exo-β-(1→3)-galactanase, β-glucuronidase and endo-β-(1→6)-galactanase. The oligosaccharide products were purified on a C_18_ cartridge (elution with 5% acetic acid) and cation exchange resin (Dowex; elution with 5% acetic acid) and were reductively aminated with [^12^C_6_]-aniline (wild-type oligosaccharides; black line) and [^13^C_6_]-aniline (*fut4*, *fut6* and *fut4/fut6*; red, blue and magenta lines respectively). The labelled oligosaccharides were purified from the reductive amination reagents on a normal phase cartridge (Glyko clean S) and the purified glycans were separated by HILIC and analysed by MALDI-ToF-MS. Although all three fucosylated oligosaccharides were detected for wild-type and *fut6* samples, no corresponding glycans were detected from AGP leaf extracts from *fut4* and *fut4/fut6* plants. Background peaks are marked with an asterisk (*). On panel b the Col-0 trace (black line) partly coincides with, and therefore obscures, the *fut6* (blue line) trace.

Recently we identified the fucosylated oligosaccharides released by AG specific hydrolases from Arabidopsis leaf AGP as *α*-L-Fuc-(1→2)-*α*-L-Ara*f*-(1→3)-*β*-Gal*p*-(1→6)-*β*-Gal*p*-(1→6)-Gal*p* (FucAraGal_3_) and *α*-L-Fuc-(1→2)-*α*-L-Ara*f*-(1→3)-*β*-Gal*p*-(1→6)-*β*-Gal*p*-(1→6)-*β*-Gal*p*-(1→6)-Gal*p* (FucAraGal_4_) [14]. An oligosaccharide composed of two pentoses, Fuc and three Gal*p* residues was also released, and we proposed that the structure of that oligosaccharide might be *α*-L-Fuc-(1→2)-*α*-L-Ara*f*-(1→?)-*α*-L-Ara*f*-(1→3)-*β*-Gal*p*-(1→6)-*β*-Gal*p*-(1→6)-Gal*p* (FucPent_2_Gal_3_) [14]. We investigated whether these fucosylated leaf AG oligosaccharides were released by digestion of AGP extracts from the *fut4*, *fut6* and *fut4/fut6* double mutants with the Oligosaccharide relative Quantitation using stable Isotope Tagging (OliQuIT) method [29]. The OliQuIT method takes advantage of the fact that the chromatographic elution positions of an oligosaccharide labelled with ^13^C_6_- or ^12^C_6_-aniline are identical. This property can therefore be used for comparison of the abundance of oligosaccharides between samples. Hence, purified AGP extracts from wild type and mutant leaves were hydrolysed with AG-specific *α*-arabinofuranosidase followed by endo-*β*-(1→6)-galactanase and the purified oligosaccharide products were reductively aminated with ^13^C_6_- or ^12^C_6_-aniline. The isotope tagged oligosaccharides were then separated by HILIC coupled off-line to MALDI-ToF-MS and the abundances of the differently labelled oligosaccharides were compared. The extracted ion chromatograms (EICs; Figure 3 a, b and c) indicate that all three of the Fuc-modified oligosaccharides are absent from *fut4* and the *fut4/fut6* double mutant but were present in *fut6* mutant leaves. Together with the monosaccharide composition data, this MS data suggests that FUT4 is solely responsible for the fucosylation of all three oligosaccharides in Arabidopsis leaf AG polysaccharides.

### Arabidopsis AGP Fucosylation differs between Leaves and Roots

**Figure 4:**
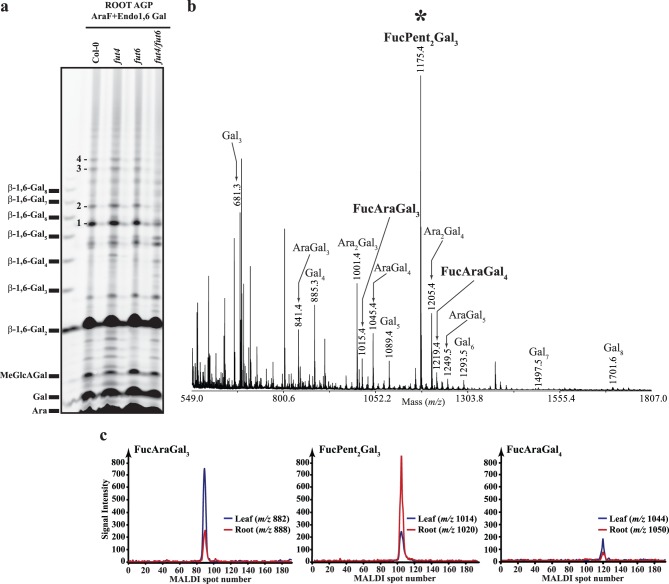
Characterisation of oligosaccharides released by the sequential digestion with the AG-specific enzymes α-arabinofuranosidase and endo-β-(1→6)-galactanase from Arabidopsis root AGP extracts. (a) Polysaccharide Analysis using Carbohydrate gel Electrophoresis (PACE). Oligosaccharide products from wild-type (Col-0), *fut4*, *fut6* and *fut4/fut6* were reductively aminated with 2-aminonaphthaline trisulfonic acid and separated by electrophoresis on acrylamide gels. An oligosaccharide ladder prepared from β-(1→6)-galactan was used as migration marker. The numbers indicate putatively fucosylated oligosaccharides with altered abundance in the wild-type and *fut* mutant samples. (b) MALDI-ToF-MS spectrum of *per*-methylated oligosaccharide products from wild-type plants. Peaks marked with an asterisk (*) were selected for high-energy MALDI-CID structural analysis. (c) Extracted ion chromatograms (EICs) for the fucosylated oligosaccharides originating from Arabidopsis leaf (blue lines) and root (red lines) AGP extracts hydrolysed sequentially by α-arabinofuranosidase, exo-β-(1→3)-galactanase, β-glucuronidase and endo-β-(1→6)-galactanase. Arabidopsis root AGP extracts contain the same three fucosylated oligosaccharides as leaves albeit in different relative abundances.

The fucosylated oligosaccharides released by digestion of root AGPs have not previously been determined. Therefore, we structurally characterised the AG oligosaccharides by PACE and MALDI-ToF-MS. Root AGP extracts were hydrolysed with α-arabinofuranosidase followed by endo-*β*-(1→6)-galactanase to remove terminal Ara modifications and to cleave the *β*-(1→6)-galactan side chains, releasing the fucosylated residues. The resulting oligosaccharides were analysed by PACE (Figure 4a). From Figure 4a it is clear that root AGP extracts are susceptible to the enzymatic treatment releasing a ladder of oligosaccharides with degree of polymerisation (DP) extending from 1 to 12. Some of the oligosaccharides that do not comigrate with *β*-(1→6)-galactooligosaccharide standards (marked with numbers 1 to 4) are substantially reduced or missing from the *fut4/fut6* double mutant suggesting that they may be fucosylated. To characterise further the released oligosaccharides, enzyme digested samples were analysed by MALDI-ToF-MS (Figure 4b). The α-arabinofuranosidase and endo-*β*-(1→6)-galactanase sequential digestion released oligosaccharides consisting predominantly of hexose (Hex), pentose (Pent) and deoxyhexose (Deoxyhex). A series of oligosaccharides corresponding to Hex_3–8_ and Pent_1–2_Hex_3–5_ were seen, and three Deoxyhex-containing oligosaccharides corresponding to DeoxyhexPent_1–2_Hex_3–4_. The hexosyl and the pentosyl residues in the Hex_3–8_ and Pent_1–2_Hex_3–5_ oligosaccharides, respectively are Gal and Ara since the oligosaccharides arise from AG-specific enzyme digestion.

To determine whether the Deoxyhex containing oligosaccharides were the same fucosylated structures found in leaf AGP, we used the comparative approach using the [^13^C_6_]aniline/[^12^C_6_]aniline labelling (OliQuIT). The HILIC-MALDI-ToF-MS EICs for FucAraGal_3_, FucAraGal_4_ and FucPent_2_Gal_3_ oligosaccharides from leaves (*m/z* 882, 1044 and 1014, respectively labelled with the light isotope [^12^C_6_]) and the root (*m/z* 888, 1050 and 1020, respectively labelled with the heavy isotope [^13^C_6_]) are shown in Figure 4c. For each of the three oligosaccharides the same main structural isomer was detected in both leaf and root tissues. However, the relative abundance of these oligosaccharides varies between leaves and roots. In particular, the FucPent_2_Gal_3_ oligosaccharide was much more abundant in roots than in leaves.

### Arabidopsis *fut4/fut6* AGPs from Roots Lack Fuc Modifications

**Figure 5:**
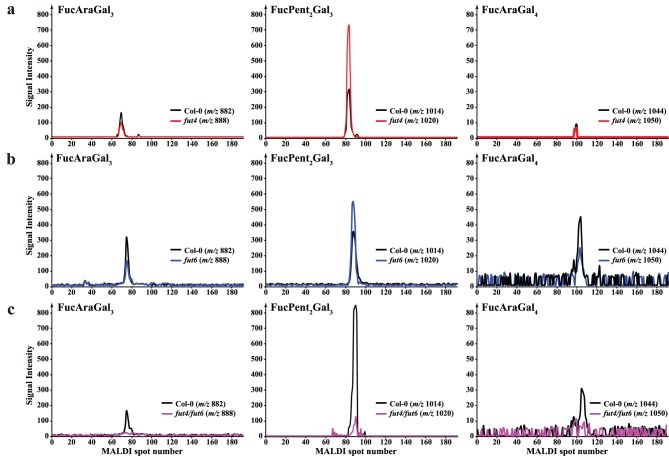
EIC for the L-Fuc modified oligosaccharides from Arabidopsis root AGP extracts of wild-type (black line), *fut4* (red line), *fut6* (blue line) and *fut4/fut6* (magenta line) plants. Although all three fucosylated oligosaccharides were detected for wild-type, *fut4* and *fut6* samples, no FucAraGal_3_ and FucAraGal_4_ were detected from AGP root extracts from *fut4/fut6* plants and very small amount of FucPent_2_Gal_3_ oligosaccharide was detectable in root AGP extracts from *fut4/fut6* plants but its abundance relative to wild-type is significantly reduced.

Since both *FUT4* and *FUT6* are expressed in Arabidopsis roots and the monosaccharide analysis from root AGP extracts indicated a reduction in Fuc levels in both *fut4* and *fut6* mutants and near absence in the *fut4/fut6* double mutant, we hypothesised that both AtFUT4 and AtFUT6 enzymes may be responsible for the fucosylation of AGPs in this tissue. Root AGPs for *fut4*, *fut6* and *fut4/fut6* double mutants were extracted and subjected as before to α-arabinofuranosidase and endo-*β*-(1→6)-galactanase sequential digestion to release the fucosylated oligosaccharides resistant to this treatment. The enzymatic hydrolysis products were compared with those prepared from the wild-type plants using the OliQuIT approach. Figure 5 shows the HILIC-MALDI-ToF-MS EICs for FucAraGal_3_, FucAraGal_4_ and FucPent_2_Gal_3_ oligosaccharides from wild-type (*m/z* 882, 1044 and 1014 respectively labelled with the light isotope) and the *fut4*, *fut6* and *fut4/fut6* mutants (*m/z* 888, 1050 and 1020 respectively labelled with the heavy isotope). Comparison of the EICs for all three oligosaccharides from wild-type and *fut4* mutants indicates a reduction in the relative abundance of FucAraGal_3_ and FucAraGal_4_ and an increase in the relative abundance of FucPent_2_Gal_3_ oligosaccharide relative to the wild-type. Similar changes were seen in *fut6* mutant samples. FucAraGal_3_ and FucAraGal_4_ oligosaccharides were absent from *fut4/fut6* samples. A small amount of FucPent_2_Gal_3_ oligosaccharide was detectable in root AGP extracts from *fut4/fut6* plants but its abundance relative to wild-type is significantly reduced. This data suggests that both *FUT4* and *FUT6* enzymes contribute to the fucosylation of Arabidopsis root AG polysaccharides, and that they are both able to generate all three fucosylated structures.

### One of the Fucosylated Oligosaccharides from Arabidopsis AGPs Contains a Xyl Residue

The reduction in Xyl from AGP extracts from the *fut4* and *fut4/fut6* mutants in leaves and *fut4*, *fut6* and *fut4/fut6* in roots, closely followed the changes in abundance of the FucPent_2_Gal_3_ oligosaccharide (Figure 2, 4). This suggests that a Xyl residue is present on the AGP in these tissues, and the addition of the Xyl is dependent on the presence of Fuc. For example, fucosylation could provide the context to either add a terminal Xyl to a side chain containing Fuc or to add a Xyl residue to the galactan backbone in close proximity to Fuc. To test these hypotheses we investigated whether Xyl was detectable in the oligosaccharide with unknown structure FucPent_2_Gal_3_. Thus root AGP extracts from wild-type plants were hydrolysed with arabinofuranosidase followed by endo-*β*-(1→6)-galactanase, the oligosaccharide products were purified and labelled with 2-AA and separated with HILIC. The fractions containing the FucPent_2_Gal_3_ oligosaccharide were collected from the HILIC column and hydrolysed by TFA. Even though very low amounts of the oligosaccharide were available, the sugar content was identified by HPAEC-PAD monosaccharide analysis. The data in Table 1 is consistent with the FucPent_2_Gal_3_ oligosaccharide composition being FucXylAraGal_3_. The mannose is likely to arise from contamination of the oligosaccharide, as there is no mannose in the oligosaccharide structure detected by NMR (see below).

**Table 1:** HILIC purified oligosaccharide: HPAEC-PAD neutral monosaccharide analysis (% Mol).

Sugar	Root oligosaccharide
Fuc	8.02±2.29
Ara	24.79±0.02
Gal	42.29±0.57
Xyl	18.05±0.59
Man	6.45±2.33

Values represent mean ± SD (n = 2).

**Figure 6:**
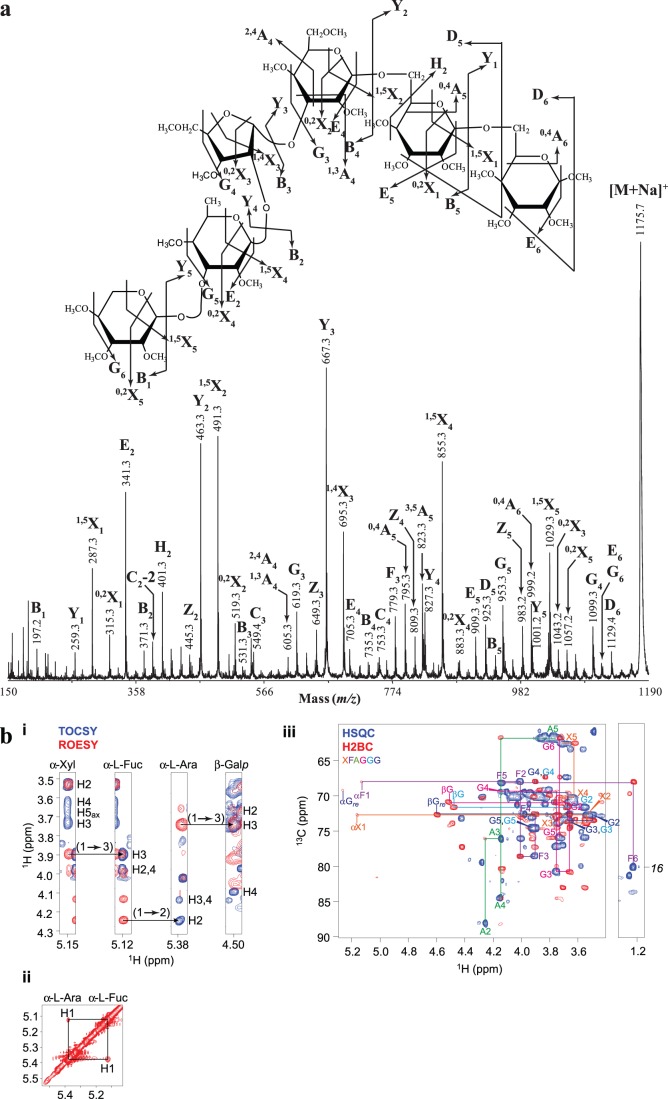
(a) Structural characterisation of the *per*-methylated FucPent_2_Gal_3_ oligosaccharide by high-energy MALDI-CID. Arabidopsis root AGP extracts were sequentially hydrolysed with α-arabinofuranosidase and endo-*β*-(1→6)-galactanase and the hydrolysis products were purified, *per*-methylated and analysed by MALDI-ToF-MS as shown in Figure 4b. The oligosaccharide with *m/z* 1175.3 as selected for MALDI-CID analysis and was identified as Xyl-(1→3)-*α*-L-Fuc-(1→2)-*α*-L-Ara-(1→3)-*β*-Gal*p*-(1→6)-*β*-Gal*p*-(1→6)-Gal*p*. Glycosidic cross-ring fragments were identified according to the Domon and Costello nomenclature (1988). (b) HPAEC-PAD monosaccharide analysis of neutral sugars for the HILIC purified root oligosaccharide. Arabidopsis root AGP extracts were sequentially hydrolysed with α-arabinofuranosidase and endo-*β*-(1→6)-galactanase and the hydrolysis products were reductively aminated with 2-AA and separated with HILIC. The FucPent_2_Gal_3_ oligosaccharide was collected from the HILIC column and hydrolysed by TFA. The sugar content was identified by HPAEC-PAD monosaccharide analysis. (b) NMR analysis of the FucPent_2_Gal_3_ hexasaccharide. (i) H-1 strip plots from 2D ^1^H-^1^H TOCSY (blue) and ROESY (red) spectra showing the NOE connectivity arising from the Xyl-(1→3)-*α*-L-Fuc-(1→2)-*α*-L-Ara-(1→3)-*β*-Gal*p* glycosidic linkages. (ii) 2D ^1^H-^1^H ROESY spectrum of the *α*-H1 region showing the Fuc H-1/Ara H-1 NOE arising from their close proximity due to the *α*-L-Fuc-(1→2)-*α*-L-Ara glycosidic linkage. (iii) 2D ^13^C HSQC and H2BC spectra showing the assignment of ^1^H,^13^C HSQC peaks using H2BC, which exclusively reveals sequential connections over two covalent bonds (Nyberg et al., 2005). The H-1 chemical shift of the non-reducing-end Xyl is consistent with an *α* configuration; the ^13^C resonance positions of Fuc C-3, Ara C-2 and Gal C-3 are downfield shifted consistent with their involvement in glycosidic linkages. (NB: Fuc C-6 was aliased in the spectra; the actual resonance frequency c. 16 ppm is shown for clarity.).

To identify the structure of the FucXylAraGal_3_ hexasaccharide, we next carried out high energy MALDI-CID. The tandem mass spectrometry (MS/MS) spectrum of the *per*-methylated FucXylAraGal_3_ (*m/z* 1175.7) oligosaccharide is shown in Figure 6a. The series of Y and ^1,5^X ions [30] reveals the sequence of the sugars on the oligosaccharide chain. Starting from the reducing end the order of sugars was as follows: Gal, Gal, Gal, Ara, Fuc and Xyl. The ^0,4^A_5_ and ^0,4^A_6_ ions, which are unique to the non-reducing end, indicate that the three Gal residues are connected via a (1→6)-linkage. The presence of the G_3_ and E_4_ [31] ‘elimination ions’ together with the ^1,3^A_4_ and ^2,4^A_4_ non-reducing end ions, indicate a linkage of the Ara at the C-3 of the third Gal residue from the reducing end. Similarly, the G_4_ ‘elimination ion’ and the ^0,2^X_3_ reducing-end ion are indicative of a branching at the C-2 of the Ara. Finally, the presence of a G_4_ ‘elimination ion’ indicated that an Ara in furanose form is present on the hexasaccharide. To study the structure further by NMR, AGPs were extracted from hydroponically grown roots and were subjected to sequential digestion with the AG-specific enzymes α-arabinofuranosidase and endo-*β*-(1→6)-galactanase. The hydrolysis products were separated on a Size Exclusion Chromatography (SEC) column, and the fractions containing the FucPent_2_Gal_3_ hexasaccharide as judged by PACE, were combined and analysed by NMR (Figure 6b). Chemical-shift assignments were obtained using 2D ^1^H-^1^H TOCSY and ROESY alongside 2D ^13^C HSQC, H2BC, HSQC-TOCSY and HSQC-ROESY experiments. The non-reducing-end Xyl residue was readily identified and the chemical shifts of the H-1 and C-1 were consistent with an *α* configuration. The (1→3) linkage to Fuc was apparent from the intense NOE from Xyl H-1 to Fuc H-3 taken together with the downfield shift of Fuc C-3 characteristic of a glycosidic link. The Fuc-(1→2)-Ara and Ara-(1→3)-Gal linkages were similarly confirmed by a combination of NOEs and downfield ^13^C chemical shifts of Ara C-2 and Gal C-3. Although the chemical-shift positions of the remaining Gal C-5/H-5 and C-6/H-6 were severely overlapped, 2D ^13^C HMBC connections were observed across the glycosidic linkages from the H-1 to the C-6, confirming the (1→6) linkages. Otherwise, the assignment was complete and is shown in Table 2. No mannose was present in this oligosaccharide, although mannose was observed as part of a separate, contaminating oligosaccharide co-purifying by SEC, along with free 4-OMe-*β*-D-Glc*p*A.

**Table 2:** ^1^H and ^13^C NMR assignments of *α*-Xyl-(1→3)-*α*-L-Fuc-(1→2)-*α*-L-Ara-(1→3)-*β*-Gal*p*-(1→6)-*β*-Gal*p*-(1→6)-Gal*p*. at 25°C in D_2_O.

Residue		Assignment					
		1	2	3	4	5	6
*α*-Xyl_nr_	^1^H	5.150	3.526	3.724	3.617	3.666, 3.698	
	^13^C	101.47	72.61	73.68	70.15	62.49	
*α*-L-Fuc	^1^H	5.119	3.995	3.894	3.964	4.128	1.211
	^13^C	98.95	67.88	78.46	72.54	68.05	16.08
*α*-L-Ara	^1^H	5.374	4.242	4.132	4.132	3.722, 3.739	
	^13^C	108.54	88.05	76.03	84.42	61.71	
*β*-Gal*p*	^1^H	4.502	3.651	3.740	4.090	3.727	3.765, 3.837
	^13^C	103.90	70.81	80.68	69.36	75.81	61.68
*β*-Gal*p*	^1^H	4.467	3.540	3.663	3.968	*∼3.901*	*∼3.915, 4.053*
	^13^C	104.01	71.52	73.37	69.40	*∼74.50*	69.86
*β*-Gal*p*_re_	^1^H	4.586	3.485	3.650	3.952	*∼3.901*	*∼3.901, 4.051*
	^13^C	97.21	72.59	73.39	69.51	*∼74.50*	70.16

The compositional data taken together with the MALDI-CID and NMR data allow the identification of the FucPent_2_Gal_3_ hexasaccharide as *α*-Xyl-(1→3)-*α*-L-Fuc-(1→2)-*α*-L-Ara-(1→3)-*β*-Gal*p*-(1→6)-*β*-Gal*p*-(1→6)-Gal*p*.

### The Fucose-deficient *fut4/fut6* Double Mutant is Salt-sensitive

**Figure 7:**
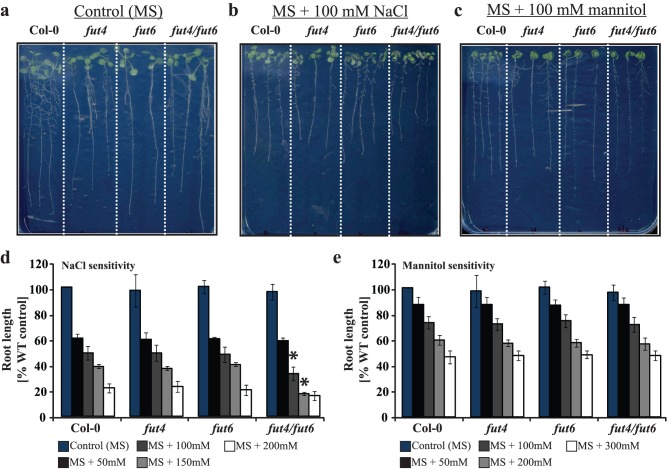
Phenotypic analysis of Arabidopsis *fut* mutants compared to wild-type plants grown on solid medium. Wild-type, *fut4*, *fut6* and *fut4/fut6* seedlings were grown on MS medium for 7 days and then transferred to cellophane layered MS-agar medium plates without any supplements (a), with 100 mM NaCl (b) and with 100 mM mannitol (c) and grown for additional 7 days. (d) *fut4/fut6* double mutants are salt sensitive. The root growth response of *fut4*, *fut6* and *fut4/fut6* mutant plants to various NaCl concentrations was compared with those of wild-type. Data are presented as percentage relative to the growth of wild-type on MS medium. Bars represent mean ± SD (n = 3). A significant difference was identified between wild type and *fut4/fut6* mutant plants as indicated by *p*>0.05 in students *t-*test. (e) *fut4/fut6* double mutants are not sensitive to osmotic stress. The root growth response of *fut4*, *fut6* and *fut4/fut6* mutant plants to various mannitol concentrations was compared with those of wild-type. Data are presented as percentage relative to the growth of wild-type on MS medium. Bars represent mean ± SD (n = 3).

The importance of AGP fucosylation in controlling root cell elongation has been proposed from studies of the Arabidopsis mutant *mur1* [32] where alterations in root elongation and morphology were observed and also with the *salt overly sensitive 5* (*sos5/fla4*) mutant which exhibited root tip swelling and arrest in root growth under salinity stress [33]. We therefore investigated whether *fut* mutants may also exhibit a root phenotype. The roots of both *fut4* and *fut6* single mutants grew comparably to the wild-type, and they did not exhibit any response to salt growth (Figure 7 a, b and d). The roots of the *fut4/fut6* double mutant also grew to the same length as the wild-type under no salt stress conditions. However, the root length of the mutant was significantly reduced compared to wild-type under salt stress conditions (100 mM and 150 mM NaCl) (Figure 7b and d). Under salt stress conditions a difference in the lateral root formation between the *fut* mutants and wild-type may also be apparent (Figure 7b), but any difference was not quantified. To investigate whether the short root phenotype was due to salt sensitivity or to the osmotic shock caused by the high salt concentration, seedlings from the wild-type, *fut4*, *fut6* and *fut4/fut6* double mutant were grown on solid MS medium supplemented with mannitol. Figure 7c and e shows that *fut4/fut6* root growth was not hypersensitive to osmotic stress caused by mannitol. Together, these findings suggest that absence of fucosylation on root AGPs causes salt sensitivity of root growth in Arabidopsis.

## Discussion

We have shown that three fucosylated oligosaccharides are released by enzymatic digestion of AG from root AGPs. These are the same structures as those previously shown in leaf AGP [14]. Our data indicate that FUT4 is required for the fucosylation of all these structures in leaf AGPs and that both FUT4 and FUT6 contribute to the fucosylation of the root AGPs.

All three fucosylated oligosaccharides (FucAraGal_3,_ FucAraGal_4_ and FucPent_2_Gal_3_) previously identified in leaves [14] were found in roots, but the relative abundance of these oligosaccharides in leaves relative to roots was different. To our knowledge, this is the first time that the AGP fucosylated structures for Arabidopsis roots are reported. We investigated which fucosylated structures are affected in *fut4*, *fut6* and *fut4/fut6* mutants. Leaf and root AGPs for *fut4*, *fut6* and *fut4/fut6* double mutants were extracted and subjected as before to α-arabinofuranosidase and endo-β-(1→6)-galactanase sequential digestion to release the fucosylated oligosaccharides resistant to this treatment. The enzymatic hydrolysis products were compared with those prepared from the wild-type plants by PACE and using the OliQuIT approach. Our analysis showed that FucAraGal_3_ and FucAraGal_4_ oligosaccharides were clearly absent from leaf *fut4*, and root *fut4/fut6* samples. A small amount of FucPent_2_Gal_3_ oligosaccharide was detectable in root AGP extracts from *fut4/fut6* plants but its abundance relative to wild-type was significantly reduced. These data suggest therefore, that the fucosylation of AGPs in leaves is entirely dependent on FUT4. In contrast, in roots fucosylation of AGPs is the result of the action of both FUT4 and FUT6 enzymes, which apparently fucosylate the same structures. The view that *AtFUT4* and *AtFUT6* are functionally non-redundant enzymes that transfer Fuc residues onto different acceptor structures on AGP molecules [27] is apparently not consistent with our findings. A possible explanation for this inconsistency may be the great heterogeneity of the AGP molecules. The glycan structure of AGPs varies greatly between different cell-types, developmental stage or species [34]. Therefore it is conceivable that the FUT6 enzyme is fucosylating a specific group of root AGPs that is not fucosylated by FUT4. However, the view that FUT4 and FUT6 are the only enzymes fucosylating AGPs [27] is consistent with our findings. Similarly to FUT4, the FUT2, FUT5, FUT7 and FUT10 proteins are expressed in leaves [28], however when *FUT4* was disrupted by the T-DNA insertion in *fut4* mutant, no compensation by the other enzymes was observed suggesting that they may not be involved in the biosynthesis of the AGP molecules.

The HPAEC-PAD monosaccharide compositional analysis of leaf and root AGPs showed not only a reduction in Fuc levels (in *fut4* and *fut4/fut6* double mutant in leaves and in *fut4*, *fut6* and *fut4/fut6* in roots) but also a concomitant reduction in Xyl levels. This coincident reduction in Xyl from AGPs from the *fut* mutants suggests that fucosylation provides the context to add a terminal Xyl residue to a side chain containing Fuc or to add a Xyl residue in close proximity to Fuc. Since the structure of the FucPent_2_Gal_3_ oligosaccharide detected both in leaves and roots, was unknown we investigated the presence of Xyl in this oligosaccharide by HPAEC-PAD. Indeed, the composition of this oligosaccharide was identified as FucXylAraGal_3_. High energy MALDI-CID combined with NMR analysis for the FucXylAraGal_3_ hexasaccharide allowed the identification of the above oligosaccharide as *α*-Xyl-(1→3)-*α*-L-Fuc-(1→2)-*α*-L-Ara*f*-(1→3)-*β*-Gal*p*-(1→6)-*β*-Gal*p*-(1→6)-Gal*p*. This finding supports the hypothesis that Fuc provides the context to add a terminal Xyl to the side chain containing the Fuc residue. To our knowledge this is the first time that the *α*-Xyl-(1→3)-*α*-L-Fuc oligosaccharide is reported for AGPs. *α*-D-Xyl*p*-(1→3)-*α*-L-Fuc*p* is found in pectin, RG-II [35] but there the Xyl residue is methylated, and Fuc is linked to a Rha residue via an *α*-(1→4)-linkage. The presence of Xyl on AGPs has been reported recently for the APAP-1 structure [15]. However, the structure reported is different, and the APAP-1 AGP is described covalently linked to short arabinoxylan.

Even though AGPs account for less than 10% of the wall matrix, they have been implicated in a number of cellular processes [12] such as programmed cell death, somatic embryogenesis [3], cell expansion, root formation and development [36]. Treatment of Arabidopsis seedlings with Yariv, a phenylglycoside that binds to the β-(1→3)-galactan backbone of AGPs [26], causes a disruption of root growth and abnormal morphology [36]. A phenotype similar to the one caused by Yariv binding has been reported for the *root epidermal bulger* (*reb-1*) mutant [37]. The *REB-1* gene encodes for a UDP D-glucose 4-epimerase that converts D-Glc to D-Gal providing Gal to AGPs [38]. This finding confirms the hypothesis that AGPs are important for the control of cell expansion in elongating roots. Further confirmation of the involvement of AGPs in controlling cell root elongation comes from the Arabidopsis mutant *mur1* which is deficient in the biosynthesis of Fuc [32]. The *mur1* mutant is lacking fucosylated AGPs in leaves [14] and roots and exhibits an alteration in root morphology and elongation that has been ascribed to AGP fucosylation [25]. However, the *fut4/fut6* mutant did not show a root phenotype under normal growth conditions suggesting that the *mur1* short root phenotype can not be attributed entirely to the lack of Fuc on root AGPs. In the *mur1* mutant, besides AGPs other Fuc-containing glycans were affected such as xyloglucan, RG-I and RG-II and N-linked glycans, therefore the root phenotype may be the effect of under-fucosylation of multiple cell wall components.

Absence of the SOS5/FLA4 protein, a putatively cell surface adhesion protein with AGP-like and fasciclin-like domains in the *sos5/fla4* mutant leads to root tip swelling and arrest in root growth [33] under salt stress conditions. This suggested that FUT mutants may also exhibit a root phenotype, and indeed salt-sensitivity assays revealed that the *fut4/fut6* double mutant has shorter roots under high-salt concentrations. Experiments on solid media supplemented with mannitol instead of NaCl at equivalent osmotic pressure showed that the short root phenotype is not due to osmotic stress but is caused by the salt. Since the short root phenotype was observed only in seedlings from the double *fut4/fut6* mutant and not in either of the single *fut* mutants, the severe reduction of Fuc on root AGPs rather than absence on a subgroup of AGPs seems to cause the phenotype. It is not possible at this time to assign the salt-sensitivity phenotype to the lack of a specific fucosylated oligosaccharide structure, since the *fut4/fut6* mutants were found to lack both the terminal fucose-containing oligosaccharides and the Xyl*p*-(1→3)-*α*-L-Fuc*p* oligosaccharide. The identification of a xylosyltransferase for AGPs may help to shed a light onto the salt sensitivity phenotype. However, these findings are suggesting that fucosylated AGPs have an important role in maintaining proper cell expansion under salt stress.

As this manuscript was being submitted, a report was published on the *fut4* and *fut6* mutants [39]. Their AGP sugar composition data is consistent with the data presented here, but that work did not address the structures of the AG oligosaccharides affected by the mutations or show the functional redundancy of FUT4 and FUT6.

## Materials and Methods

### Plant Material, Preparation of AG Proteins and AG Specific Enzymes

Arabidopsis (*Arabidopsis thaliana*) seeds were surface sterilised and sown on soil as previously described [14]. Six weeks post stratification healthy green leaves from the rosettes were collected and frozen immediately in liquid nitrogen and then stored in −80°C before further processing. Tissue used to isolate AGPs from roots was collected from six week old plants grown hydroponically [40], frozen in liquid nitrogen and stored in −80°C. Arabidopsis leaf and root AGPs were prepared using a previously established protocol [14]. For complementation analysis the AGPs were prepared as described above and were precipitated with the addition of 2 mg ml^−1^ (*β*-D-Glc)_3_-Yariv reagent made according to [14]. The Yariv-AGP pellet was “salted-out” according to [41], [42] before being dried and analysed for sugar composition. When AGPs were precipitated by Yariv the reagent was not removed from the AGPs and as a result glucose was not included in the analysis. *α*-L-Arabinofuranosidase (EC 3.2.1.55); exo-*β*-(1→3)-galactanase (EC 3.2.1.145); *β*-glucuronidase (EC 3.2.1.31) and endo-*β*-(1→6)-galactanase (EC 3.2.1.164) were prepared by methods described previously [43], [44], [45], [46].

### Identification of T-DNA by PCR and RT-PCR

Seeds of T-DNA insertional lines SAIL_284_B05 (*fut4*), SALK_078357 (*fut6*) and the *fut4/fut6* double knock out construct were obtained from NASC (Nottingham, UK). Plants homozygous for the insertion were detected by PCR and the corresponding primer sequences and PCR conditions are listed in Table S1. To analyse transcript levels of *FUT4* and *FUT6*, RNA was extracted using the Qiagen PNeasy kit according to the manufactures instructions. The RT-PCR primers are listed in Table S1.

### Complementation

A genomic 7.8 Kb Pstl fragment from Bac clone F26H6 containing 2.773 kb upstream the *FUT4* coding sequence and 3.574 kb downstream was cloned into the plant transformation vector pCAMBIA 1300 and introduced into *Agrobacterium* (Agl-O). Constructs were confirmed by PCR and restriction analysis both in *E. coli* and in *Agrobacterium*. Homozygous mutant *fut4* plants were transformed with either the genomic rescue construct or the empty vector plasmid and transgenic seeds were selected on hygromycin, The genotypes of the T1 plants were confirmed by PCR (rescued plants were positive for the presence of the *fut4* T-DNA insertion in the genomic copy whereas the plants containing the empty vector only showed the presence of the T-DNA insertion). T2 seeds from individual lines were screened on hygromycin selective plates to estimate the number of insertions. Resistant seedlings from the empty vector line and seven genomic rescue lines were transplanted to soil and rosette leaves were harvested from plants of each line for AGP isolation and subsequent sugar analysis.

### Enzymatic Hydrolysis and PACE Analysis

Arabinogalactan peptide preparations (1 mg) were digested with the AG-specific enzymes as described by Tryfona *et al*. [14]. The derivatization of carbohydrates was performed according to previously developed protocols [47]. Carbohydrate electrophoresis and PACE gel scanning and quantification was performed as described by Goubet *et al.* [47]. Control experiments without substrates or enzymes were performed under the same conditions to identify any non-specific compounds in the enzymes, polysaccharides/cell walls or labelling reagents.

### AGP Oligosaccharide Sample Desalting and Clean-up

Following the enzyme digestions and prior to *per*-methylation released peptides and enzymes were removed using reverse-phase Sep-Pak C_18_ cartridges (Waters) as described previously [7]. Briefly, the AG oligosaccharides were eluted with 3 ml of 5% acetic acid and were lyophilised. Dry samples were dissolved in 0.5 ml of 5% acetic acid and were desalted using 2 ml Dowex beads (50×8, H^+^ form, 50–100 mesh) as previously described [48]. Purified samples were lyophilised.

### *Per*-methylation of Arabinogalactan Polysaccharides

*Per*-methylation of glycans was performed according to previously established protocols [49]. *Per*-methylated samples were resuspended in 100 μl MeOH and were kept at room temperature for MALDI-ToF-MS analysis.

### MALDI-ToF/ToF-MS/MS

*Per*-methylated samples were analysed by MALDI-ToF/ToF-MS/MS (4700 Proteomics Analyser, Applied Biosystems, Foster City, CA, USA) as previously described [50], using 2,5-dihydroxybenzoic acid (2,5-DHB) matrix (10 mg ml^−1^ dissolved in 50% MeOH). The above tandem mass spectrometer uses a 200 Hz frequency triple Nd-YAG laser operating at 355 nm wavelength. High energy MALDI-CID spectra were acquired with an average 10,000 laser shots/spectrum, using a high collision energy (1 kV). The oligosaccharide ions were allowed to collide in the CID cell with argon at a pressure of 2×10^−6^ Torr.

### Reductive Amination of AG Oligosaccharides and Purification

The AG oligosaccharides were reductively aminated with 2-aminobenzoic acid (2-AA, Sigma) or ^12^C_6_- and ^13^C_6_-aniline (Sigma) using optimised labelling conditions described previously [29]. The saccharides were then purified from the reductive amination reagents using a Glyko Clean S cartridge (Prozyme, San Leandro, CA) as described previously [48]. When isotopic labelling was performed samples labelled with the two aniline isotopes were mixed equally prior to purification from the reductive amination reagents.

### HILIC-MALDI-ToF/ToF-MS/MS

Capillary HILIC was carried out using an LC-Packings Ultimate system (Dionex, CA, USA) equipped with an amide-80 column (300 μm×25 cm; 3 μm particle size; Dionex) as previously described [14]. The column eluent passed through a capillary UV detector (set at 254 nm) to the MALDI sample spotter. For HILIC-MALDI-ToF/ToF tandem mass spectrometry a Probot sample fraction system (Dionex) was employed for automated spotting of the HPLC eluent onto a MALDI target at 20 s intervals. After air drying, the sample spots were overlaid with 2,5-DHB matrix and analysed by MALDI-ToF-MS. The MS spectra were obtained in automatic mode with an average 1500 laser shots/spectrum. The oligosaccharide molecular ions [M+Na]^+^ were identified in the MALDI data and their HILIC elution positions were determined by carrying out an extracted ion chromatogram (EIC). High energy MALDI-CID spectra were obtained as described above.

### Purification of XylFucAraGal_3_ Oligosaccharide by Size Exclusion Chromatography (SEC)

Root AGPs were extracted from hydroponically grown plants as described above and were subjected to sequential enzyme hydrolysis with α-arabinofuranosidase and endo-*β*-(1→6)-galactanase. SEC was performed on a gravity driven BioGel P-2 (190×2.5 cm; BioRad) column. 2 ml of concentrated sample were applied onto the column and eluted with distilled water; 2 ml fractions were collected and analysed by PACE as described above. The fractions with the XylFucAraGal_3_ oligosaccharide were combined, freeze dried and resuspended in 0.6 ml deuterium oxide for NMR analysis.

### NMR Analysis

NMR spectra were recorded at 298 K with a Bruker AVANCE III spectrometer operating at 600 MHz equipped with a TCI CryoProbe. Two-dimensional ^1^H-^1^H TOCSY, ROESY, ^13^C HSQC, H2BC, HMBC, HSQC-TOCSY and HSQC-ROESY experiments were performed, using established methods [51], [52]; the mixing times were 70 ms and 200 ms for the TOCSY and ROESY experiments, respectively. Chemical shifts were measured relative to internal acetone (δH = 2.225, δC = 31.07 ppm). Data were processed using the Azara suite of programs (v. 2.8, copyright 1993–2014, Wayne Boucher and Department of Biochemistry, University of Cambridge, unpublished) and chemical-shift assignment was performed using Analysis v2 [53].

### TFA Hydrolysis and HPAEC-PAD Monosaccharide Analysis

Samples were hydrolysed in 2 M trifluoroacetic acid (TFA) for 1h at 120°C. Following evaporation under vacuum for the removal of TFA, the samples were resuspended in 200 μL water and the monosaccharide sugars were separated using protocols adapted from Currie and Perry [54] on a Dionex ICS3000 system equipped with a PA20 column, a PA20 guard column and a borate trap (Dionex) [14].

### Growth Assay for Salt Stress Tolerance

Salt sensitivity assays were performed as described by Kang *et al.* [55]. Briefly, surface sterilised seeds were sown onto cellophane membrane (BioRad), placed on solid medium containing 0.5x Murashige and Skoog (MS) [56] salts including vitamins (Sigma) and sucrose (1% w/v), stratified for 48h at 4°C and then incubated at 21°C for 7 days. The membranes with seedlings were transferred onto MS medium supplemented with NaCl (50, 100, 150 and 200 mM) or mannitol (50, 100, 200 and 300 mM) and the plates were incubated vertically. Root growth was scored 7 days later. Plants were scanned using an Epson document scanner and root lengths were determined by using Image J software.

## Supporting Information

Figure S1 **FUT T-DNA insertional mutants.** (A) Modified image from TAIR showing the locations of T-DNA insertions within the *FUT* genes. The arrows represent the location of the primers and the orange triangles represent the T-DNA insertions. The *fut6* line was found to contain two T-DNA insertions in opposite directions. Flanking regions of the left border of the T-DNA insertion were amplified with the corresponding primer (left border primer, LB) and *fut4-* and *fut6-*specific right primers (4RP and 6RP, respectively). Similarly, PCRs with the T-DNA left borders and the *fut4-* and *fut6-*specific left primers (4LP and 6LP, respectively) were performed. (B) Confirmation by PCR that *fut4* and *fut6* genes are disrupted by the T-DNA insertions.
(EPS)

Table S1 **Primers and PCR/RT-PCR conditions used in this study.**
(PDF)

Table S2 **Complementation of the fut4 mutant: HPAEC-PAD neutral monosaccharide analysis (% Mol) of leaf AGPs.** Values represent mean ± SD (n = 2).
(PDF)
