# Supplementary material for: Characterisation of FUT4 and FUT6 α-(1→2)-Fucosyltransferases Reveals that Absence of Root Arabinogalactan Fucosylation Increases Arabidopsis Root Growth Salt Sensitivity
Source: PLoS One. 2014 Mar 25;9(3):e93291. doi: 10.1371/journal.pone.0093291 (PMC3965541; doi:10.1371/journal.pone.0093291)
Supplement: Table S1 — Primers and PCR/RT-PCR conditions used in this study. (PDF) [file pone.0093291.s002.pdf]

Table S1. Primers and PCR/RT-PCR conditions used in this study

Primers and conditions	
**Primer for PCR**	
FUT4 LP	ACA TGT TTT CAG AGC GAG CAA ATA TGA CG
FUT4 RP	CGT TTT AGA CAA CTA AAT CTT TCT ATG CAG
FUT6 LP	TTC TTG TTG CTA CTT CCA ACG GTA AAT AA
FUT6 RP	AGA GTA AGT ATT CTG TTT CCA AGC CCG TA
Garlic/SAIL insertion	GCA TCT GAA TTT CAT AAC CAA TCT CGA T
SALK insertion	TTT TTC GCC CTT TGA CGT TGG AG
**Primer for RT-PCR**	
FUT4 LP	CCG GCG AAG TTA TCA AGG GTT
FUT4 RP	AAA GGA ACA ACT TTC CCC GA
FUT6 LP	ACG ACT TCA ACA ACC AAC TTC TT
FUT6 RP	GCC CCA AAC TTG ATT CGT CG
GAPDH LP	TGC AAT CCC AGC CTT GGC ATC G
GAPDH RP	TAA CTG CCT TGC TCC TCT TGC CAA G
**PCR conditions for all reactions**	
94 ˚C – 2 min	
(94 ˚C – 15 s, 55 ˚C – 30 s, 68 ˚C – 3 min) × 15 cycles	
(94 ˚C – 15 s, 55 ˚C – 30 s, 68 ˚C – 3 min + 5 s per cycle) × 25 cycles	
68 ˚C – 10 min	

Sequences are given 5’ → 3’ (unless otherwise stated).
