# Supplementary material for: Characterisation of FUT4 and FUT6 α-(1→2)-Fucosyltransferases Reveals that Absence of Root Arabinogalactan Fucosylation Increases Arabidopsis Root Growth Salt Sensitivity
Source: PLoS One. 2014 Mar 25;9(3):e93291. doi: 10.1371/journal.pone.0093291 (PMC3965541; doi:10.1371/journal.pone.0093291)
Supplement: Table S2 — Complementation of the fut4 mutant: HPAEC-PAD neutral monosaccharide analysis (% Mol) of leaf AGPs. Values represent mean ± SD (n = 2). (PDF) [file pone.0093291.s003.pdf]

Table S2. Complementation of the *fut4* mutant: HPAEC-PAD neutral monosaccharide analysis (% Mol) of leaf AGPs. Values represent mean ± SD (n=2).

**Sugar**	**Wild type (Col-0)**	***fut4* mutant**	***fut4*; *FUT4* #9**	***fut4*; *FUT4* #16**
Fuc	3.7 ± 1.3	ND[a]	5.6 ± 0.9	6.0 ± 1.2
Rha	1.6 ± 0.1	1 ± 0.2	0.8 ± 0.1	0.9 ± 0.2
Ara	26.5 ± 2.6	33.2 ± 3.4	27.2 ± 1.6	26.6 ± 1.1
Gal	66.7 ± 3.6	65.8 ±3.1	64.0 ± 2.4	64.3 ± 2.5
Xyl	1.5 ± 0.2	ND[a]	2.4 ± 0.1	2.1 ± 0.1
Man	ND[a]	0.2[b]	0.2 [b]	0.1 [b]

[a] not detected; [b] detected in only one of the two samples.
